# Real estate announcements monitoring dataset for Latvia 2018

**DOI:** 10.1016/j.dib.2019.105064

**Published:** 2019-12-31

**Authors:** Valerijs Skribans, Māris Jurušs, Maryna Demianchuk, Natalia Maslii, Dickson Pastory

**Affiliations:** a Riga Technical University, Latvia; b Odessa Mechnikov National University, Ukraine; c College of Business Education, Tanzania

**Keywords:** Real estate, Announcements, Monitoring, Data mining, Econometrics

## Abstract

The dataset represents a collection of real estate announcements published in 2018 in the Latvian leading advertisement website www.ss.com [1]. In the Latvian case, mentioned advertisement website is alternative information source in contrast with several (5–7) large real estate agencies. The mentioned advertisement website has no important competitors in Latvia, closer competitor reklama. lv [2] is 4–5 times smaller. Advertisement website www.ss.com represents information from small and medium size agencies, as well from individuals, who want to take part in the real estate market. The collected dataset reflects the observation dynamics of 12 months during 2018, including in total 238 thousand observations. Dataset has 24 dimensions, such as in announcement mentioned price for real estate, deal types, dimensions of location of real estate, such as region, district, address; characteristics of real estate, such as real estate type (land, flat and so on), size and main characteristics for each real estate type, such as land area or bad rooms in apartments. The dataset is hosted in Data Archiving and Networked Services (DANS) repository [3].

**Table:** Specifications Table

Subject	Business, Management and Accounting (General)
Specific subject area	Business and management, marketing, business intelligence, econometrics
Type of data	Table
How data were acquired	Market announcements in internet downloading with Data Sciences technologies, data mining part. Instruments: for data collection used R Studio software, Rcrawler library Model and make of the instruments used: authors developed model (machine code) published in this article
Data format	Raw (not Analyzed and not Filtered, published as it is, grouped according its extraction process in 12 groups according each months extraction)
Parameters for data collection	The dataset represents collection of real estate announcements published in 2018 in Latvian leading advertisement website www.ss.com [1]. Data was groped by months according the collection process in one table from each observations month, no changes were made to the data.
Description of data collection	The data represent not centralized part (“grey” and “black” parts together) of real estate market offers in Latvia. In Latvia there are not a lot of announcements servers in the web. The mentioned advertising web has extremely big influence on economy of Latvian Republic and on real estate market, as well as on others sectors such as used-car market.
Data source location	City/Town/Region: Riga Country: Latvia, European Union
Data accessibility	Repository name: Data Archiving and Networked Services (DANS) repository Data identification number: Skribans, Dr.oec. V. (Riga Technical University) (2019): Latvian Real Estate Announcements Monitoring in 2018. DANS. https://doi.org/10.17026/dans-2z3-fx28 Direct URL to data: https://easy.dans.knaw.nl/ui/datasets/id/easy-dataset:117557/tab/2#

**Table:** 

**Value of the Data** • In Latvia the State Revenue Service (a direct administrative authority under the supervision of the Minister of Finance, which ensures the accounting of tax payments and taxpayers) developed a methodology and monitors Latvian leading advertisement website www.ss.com. The dataset is developed based on independent advertisement website monitoring executed by Riga Technical University. The dataset can help to check and correct the State approach in announcements monitoring, as well as to improve the State policy in real estate market. • The dataset is very interesting for Data Scientists and statistical specialists, as well as for political data analysis specialists. Recently in the internet grow amount of not correct information, which is known as “fake news”. Probably, fry advertisement websites it is possible to influent on market, with “fake announcements”. For Data Scientists and other researchers it is good challenge do develop “fake announcements” criteria and use it later in other fields. • The author used the dataset to estimate infrastructure objects in Riga (such as schools, shops and public transport stops) influence on sellers his owned object evaluation, as well as for flats from serial buildings price index development in different dimensions. • The dataset is developed based on of real estate market announcements monitored during 2018. The dataset combines different deal types: real estate sells offers, rental offers; by different regions and by different types of properties. The dataset will be very helpful for real estate market specialists. • The data is original, collected by author. Take into account, that announcements average life cycle is 4 week (after it, announcements are deleted from internet), it is not possibly repeat collection for 2018 year, so the dataset is unique, research cannot be repeated. However, the scientific protocol for collecting the data, published in this article, allow to download actual data. Based on actual data it is possible check scientific quality of data collection in 2018, as well as understand market changes in time.

## 1 Data description

Database consists of Latvian real estate announcements, total 238 th. observations. The dataset include 24 dimensions (fields). The database fields are: observations month, real estate 7 chapters (for example flats, land and so on), 18 sub chapters, deal types (such as selling offer, for rent), price (euro), 4 price units (per day, month and so on), region, district, address, rooms, area, area units, floor, floors in living building, elevator option in building, building series name, building type, facilities, floors in building, rooms in building, land area, land area units, amenities and description, land purpose.

Table 1 represent dataset observations by chapters and deal type.

**Table 1:** Announcements by real estate chapters and deal type in Latvia in 2018 (full data is available in dataset [3]).

Chapter	Deal type					
	Sell	For rent	Other	Exchange	Unknown	**Total**
Flats	67 519	26 532	112	428		**94 591**
Land	48 744	1 309	129	89		**50 271**
Houses, summer houses	38 315	3 780	696	236		**43 027**
Complex of rooms	13 919	15 409	188	45	13	**29 574**
Offices	1 747	10 834	13	2		**12 596**
Farms, homstads	5 267	144	48	69		**5 528**
Forest land	2 016		48			**2 064**
**Total**	**177 527**	**58 008**	**1 234**	**869**	**13**	**237 651**

**Fig. 1:**
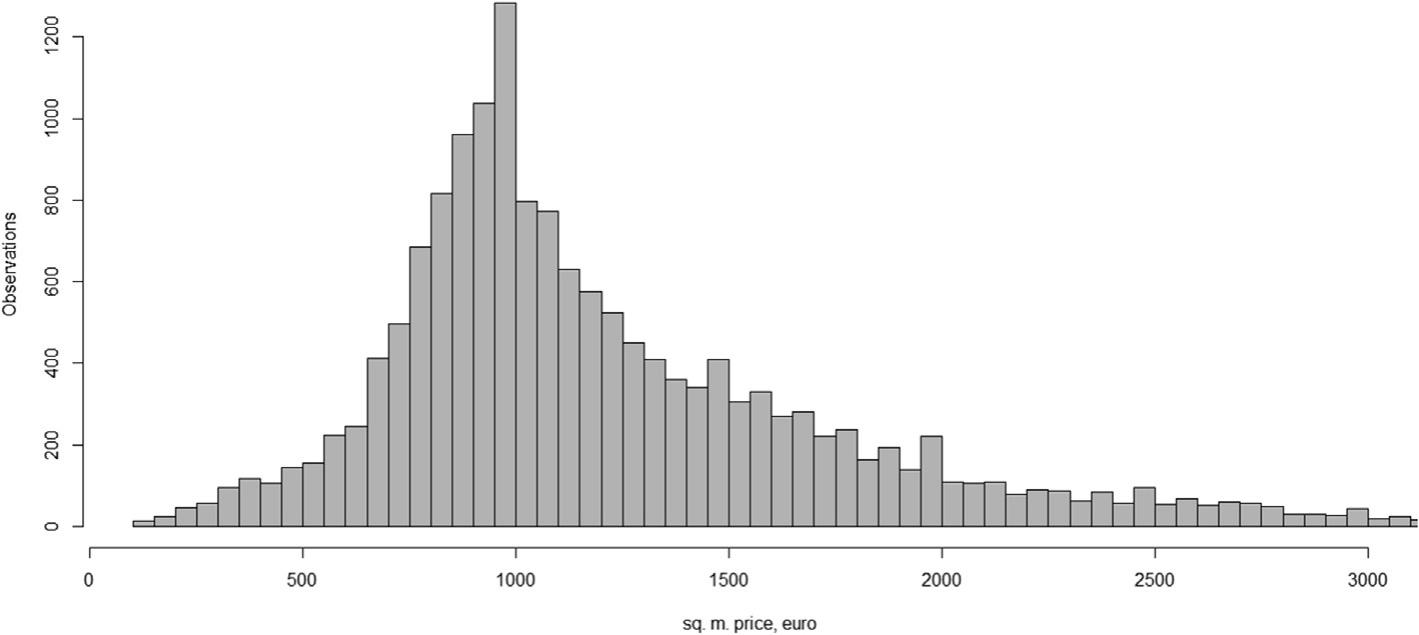
Offer prices for flats in Riga in 2018 (full data is available in dataset [3]).

In the author's opinion, the most important data field is price of real estate. Dataset includes prices from different object groups, such as land, buildings, flats. It is not possible to compare prices in different groups, but possible inside groups. Fig. 1 shows sq. m. price analysis for flats group in Riga.

**Fig. 2:**
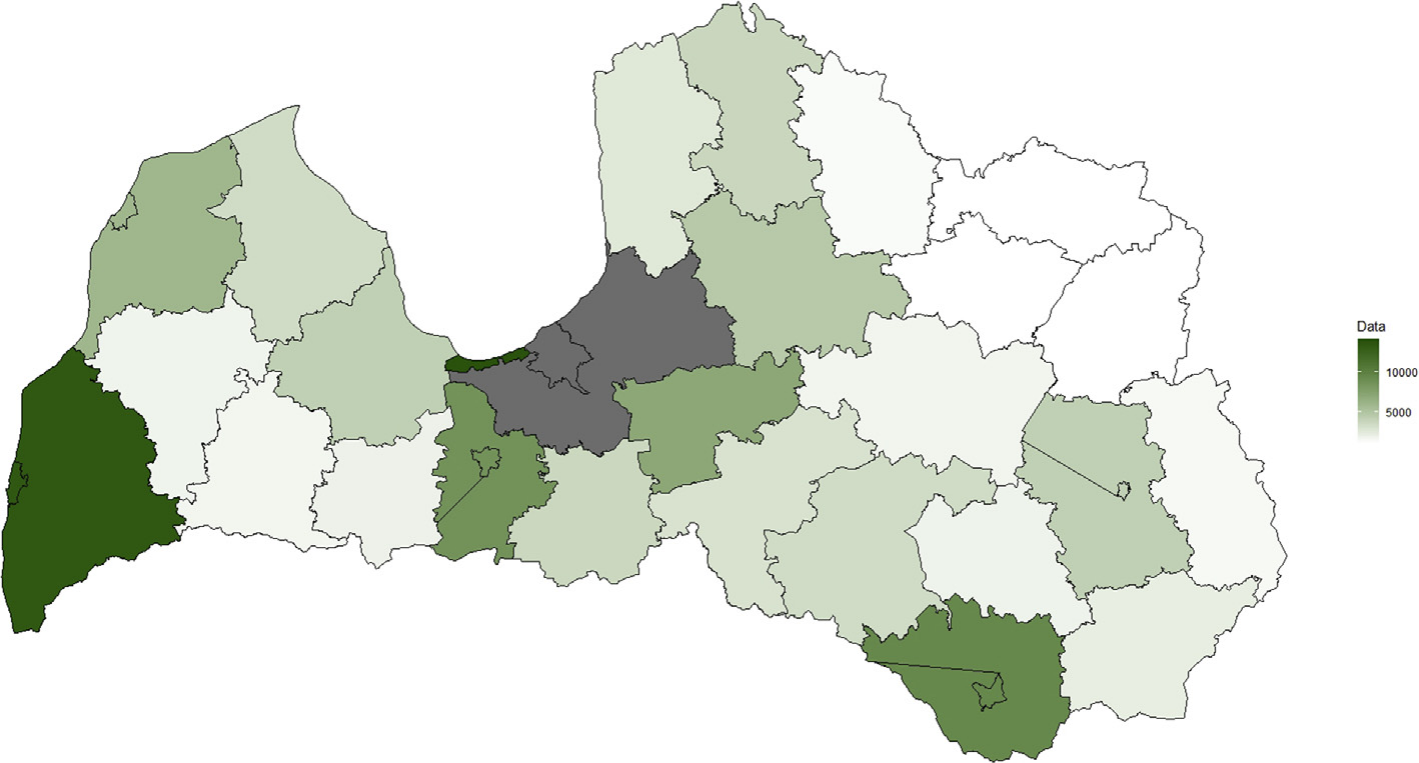
Collected real estate announcements by object allocation by Latvian districts, 2018, observations (full data is available in dataset [3]).

**Fig. 3:**
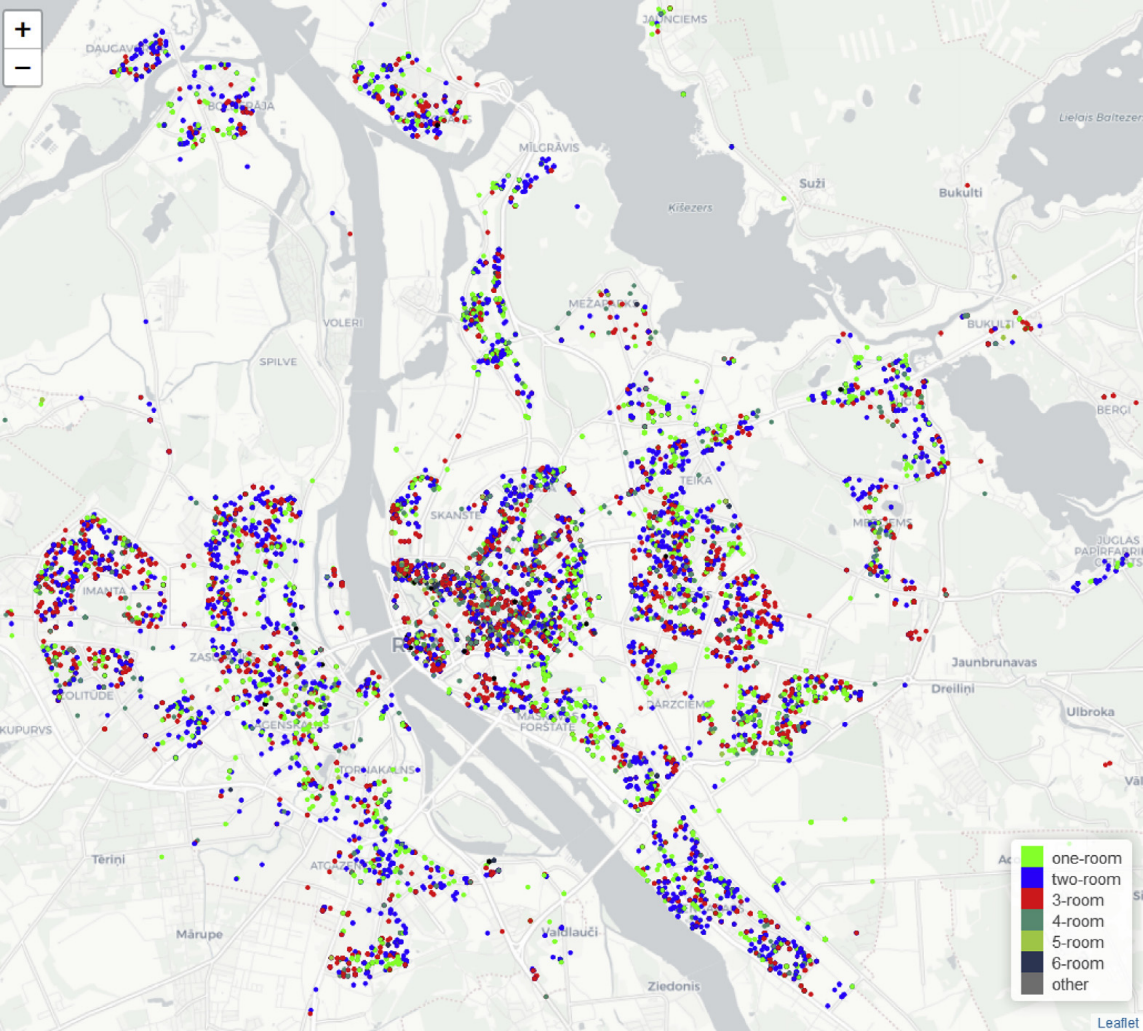
Allocation of flats offered for sale in Riga in 2018, by rooms in flat (full data is available in dataset [3]).

Fig. 2 represent collected real estate announcements by Latvian districts. Riga district have 38.2 th. observations (14% of all observations), but capital city of Latvia, Riga have 90 th. observations (33% of all observations), its reason, why Riga city real estate is analyzed separately, in Fig. 3.

Next important indicator are location of real estate and building series. Data of this two dimensions are shown in Table 2.

**Table 2:** Most offered for sale flats by districts and buildings series in Riga in 2018, announcements (observations) (full data is available in dataset [3]).

District	Building series type													
	103	104	119	467	602	Hrusc.	Jaun.	LT proj.	M. gim.	P. kara	Renov.	Specpr.	Stalina	**Total**
Agenskalns	7			9	4	140	164	25	14	249	107	58	34	**811**
Aplokciems							34	1					1	**36**
Bergi	7					4	53					10	1	**75**
Bierini							8			12	1	6		**27**
Bolderaja	38				8	33		32	8	28	9	52	16	**224**
Centrs	21			7		139	790	8	6	2075	949	233	161	**4389**
Ciekurkalns	4					6	43		14	40	14	25	9	**155**
Cits	1			2	1		5			3	2	4	3	**21**
Darzciems	34			1	23	3	53		27	4		23	4	**172**
Daugavgriva	50	2				22			3	3		38	5	**123**
Dzeguzkalns	1			5		19	59	63	5	18	4	16	2	**192**
Ilguciems	8		11	12	5	133	28	175	14	24	1	48	34	**493**
Imanta	1	4	2	9	314	1	225	229	3	8	7	33	2	**838**
Jaunciems	1					10			5	9		6	7	**38**
Jugla	2		1			142	99	82	13	13	19	96	14	**481**
Kengarags		1		56	1	146	72	474	24	10	26	44	30	**884**
Kipsala							38			7	17	2		**64**
Kliversala	2					9	36			16	10	11		**84**
Krasta r-ns		10		63	4	21	2	2	1	24	2	21	17	**167**
Mangali				4		15	5	9		7	3	5	15	**63**
Maskavas priekspilseta	6			4		15	9	2		197	15	20	23	**291**
Mezaparks	4			1	1	12	68	6		22	8	66	8	**196**
Mezciems	15	17	1		107	7	177	28		4		8	11	**375**
Plavnieki	7	29	46	36	426	7	134		133	1	5	32	6	**862**
Purvciems	50	59	151	76	163	88	288	231	87	12	34	106	34	**1379**
Sampeteris-Pleskodale	7					46	103	1	1	18	7	36	1	**220**
Sarkandaugava	30				6	92	41	5	6	114	42	111	34	**481**
Teika	23					72	114	8	13	69	59	102	76	**536**
Tornakalns	1					6	34	3	15	67	7	11	3	**147**
Vecaki							22			6	2	3		**33**
Vecmilgravis	32	1		13	55	43	12	125	25	18	47	38	44	**453**
Vecriga							59			134	41	3	6	**243**
Ziepniekkalns	14	4	141		143	68	262	12	23	34	12	52	13	**778**
Zolitude		15	264	1			114			2	1	8	1	**406**
**Total**	**378**	**142**	**618**	**299**	**1263**	**1311**	**3189**	**1522**	**442**	**3267**	**1456**	**1347**	**621**	**15 855**

It is important to note, that Table 2 represents only flats the most offered for sale, dataset contains also districts with less then 20 observations per district, and building series with less then 100 observation. Taking into account that so small amount of observation, this data (total 222 observations) was removed from Table 2. Dataset include all data. From Table 2 it is visible, that for scientific analysis it is needed to clear data from districts with small then 100 observations and building series with less then 150 observations.

## 2 Experimental design, materials, and methods

In Latvia it is possible to observe market advertisements in internet with Data Mining technologies. There was collected average 20 thousands offers per months of real estate in Latvia in 2018 (from 16.5 th. in January to 22.3 th. in May). After data cleaning, removing of duplication, “fake announcements” and mistakes removing, amount of observation was compared with official deals statistics data published by The State Land Service [4]. Collected announcements approximately two times (6932/15 500) exceed amount of real deal. However, average prices, take into account, that dataset collect offer price, have not so big and important difference. This means that for each real deal there are several announcements (approximately two). Another reason of difference in data may be The State Land Service data aggregation methodology, they aggregate data from February to February for the year. The State Land Service publish only aggregated data for free, does not publish information about each deal. Data mining allows to get more detailed information. This shows the advantage of the Data Science and Data Mining methods for collecting and providing information for different purposes.

The database was collected from biggest in the Latvia advertisement website www.ss.com [1] from its real estate section, by monthly repeated following R studio data scraping code (the scientific protocol for collecting the data) (The mentioned advertisement website has no important competitors in Latvia, closer competitor www.reklama. lv [2] is 4–5 times smaller):

library (Rcrawler).

Rcrawler (Website = “https://www.ss.com/lv/real-estate/”, KeywordsFilter = c (“real-estate”), no_cores = 4, no_conn = 4, 12, ExtractXpathPat = c (

″//*[@id = 'tdo_8']″,

″//*[@id = 'tdo_20']″,

″//*[@id = 'tdo_856']″,

″//*[@id = 'tdo_11']″,

″//*[@id = 'tdo_1']″,

″//*[@id = 'tdo_3']″,

″//*[@id = 'tdo_4']″,

″//*[@id = 'tdo_6']″,

″//*[@id = 'tdo_2']″,

″//*[@id = 'tdo_7']″,

″//h2 [@class = 'headtitle']″,

″//*[@id = 'tdo_57']″,

″//*[@id = 'tdo_58']″,

″//*[@id = 'tdo_60']″,

″//*[@id = 'tdo_59']″,

″//*[@id = 'tdo_228']″))

After downloading data, dataset was created with standard R Studio functions.

Riga Technical University (RTU) is one of the regional leading in quantitative economy analysis and mathematical modeling. Internet announcements monitoring was started in RTU in the end of 2017 as part of integration of new data sciences technologies in learning process. In additional, RTU researchers develop different approaches and models for transport [5,6], logistics [[7], [8], [9]] gas market [10,11], tax and duty polices [12] and medicine [13,14].
